# The Dlt and LiaFSR systems derepress SpeB production independently in the *Δpde2* mutant of *Streptococcus pyogenes*

**DOI:** 10.3389/fcimb.2023.1293095

**Published:** 2023-11-13

**Authors:** Sabrina Faozia, Tasmim Hossain, Kyu Hong Cho

**Affiliations:** Department of Biology, Indiana State University, Terre Haute, IN, United States

**Keywords:** *Streptococcus pyogenes*, c-di-AMP, phosphodiesterase, Pde2, Dlt operon, D-alanylation, teichoic acid, LiaFSR

## Abstract

The second messenger molecule, c-di-AMP, plays a critical role in pathogenesis and virulence in *S. pyogenes*. We previously reported that deleting the c-di-AMP phosphodiesterase gene *pde2* severely suppresses SpeB production at the transcriptional level. We performed transposon mutagenesis to gain insight into the mechanism of how Pde2 is involved in SpeB regulation. We identified one of the genes of the *dlt* operon, *dlt*X, as a suppressor of the SpeB-null phenotype of the *Δpde2* mutant. The *dlt* operon consists of five genes, *dltX, dltA, dltB, dltC*, and *dltD* in many Gram-positive bacteria, and its function is to incorporate D-alanine into lipoteichoic acids. DltX, a small membrane protein, is a newly identified member of the operon. The in-frame deletion of *dltX* or insertional inactivation of *dltA* in the *Δpde2* mutant restored SpeB production, indicating that D-alanylation is crucial for the suppressor phenotype. These mutations did not affect the growth in lab media but showed increased negative cell surface charge and enhanced sensitivity to polymyxin B. Considering that *dlt* mutations change cell surface charge and sensitivity to cationic antimicrobial peptides, we examined the LiaFSR system that senses and responds to cell envelope stress. The Δ*liaR* mutation in the *Δpde2* mutant also derepressed SpeB production, like the *ΔdltX* mutation. LiaFSR controls *speB* expression by regulating the expression of the transcriptional regulator SpxA2. However, the Dlt system did not regulate *spxA2* expression. The SpeB phenotype of the Δ*pde2*Δ*dltX* mutant in higher salt media differed from that of the Δ*pde2*Δ*liaR* mutant, suggesting a unique pathway for the Dlt system in SpeB production, possibly related to ion transport or turgor pressure regulation.

## Introduction

Bacteria and archaea utilize second messenger cyclic nucleotides to sense and respond to changes in their environment (Pesavento and Hengge, 2009; Kalia et al., 2013; Hengge et al., 2016; Huynh et al., 2016). These cyclic nucleotides function as signaling molecules that relay the signals by interacting with their target proteins or riboswitches in response to external or internal stimuli (Commichau et al., 2019). Several specific mono- or dinucleotides are used as second messenger molecules including cyclic adenosine phosphate (cAMP), guanosine tetraphosphate or pentaphosphate ((p)ppGpp), cyclic di-guanosine monophosphate (c-di-GMP), cyclic guanosine monophosphate-adenosine monophosphate (cGAMP), and cyclic di-adenosine monophosphate (c-di-AMP) (Corrigan and Gründling, 2013).

c-di-AMP is a second messenger molecule that is produced exclusively by prokaryotes. It is primarily found in most Gram-positive bacteria, including *Staphylococcus aureus*, *Listeria monocytogenes*, *Mycobacterium tuberculosis*, and *Streptococcus* spp., as well as certain Gram-negative bacteria such as *Chlamydia trachomatis* and *Borrelia burgdorferi* (Witte et al., 2008; Pesavento and Hengge, 2009; Woodward et al., 2010; Corrigan et al., 2011; Kamegaya et al., 2011; Barker et al., 2013; Gándara and Alonso, 2015; Andrade et al., 2016). c-di-AMP is involved in various cellular processes, including osmoregulation, DNA repair mechanism, maintenance of cell wall homeostasis, fatty acid synthesis, virulence regulation, and biofilm formation (Witte et al., 2008; Pesavento and Hengge, 2009; Woodward et al., 2010; Corrigan et al., 2011; Kamegaya et al., 2011; Barker et al., 2013; Gándara and Alonso, 2015; Andrade et al., 2016). Despite its involvement in crucial cellular processes and virulence regulation, the detailed mechanism by which c-di-AMP controls these functions is still poorly understood.

The model organism used in this study, *S. pyogenes* or Group A Streptococcus (GAS) is an obligate human pathogen that causes diverse diseases from mild superficial infections to severe invasive, toxigenic, or post-streptococcal autoimmune sequelae (Walker et al., 2014). GAS is still a significant public health concern in developed and developing countries. Approximately 700 million people worldwide suffer from pharyngitis caused by GAS each year. Inadequate treatment or repeated GAS infections develop non-suppurative autoimmune sequelae, acute rheumatic fever (ARF), which can further damage the heart and cause rheumatic heart diseases (RHD) showing high mortality (Carapetis et al., 2005). Approximately 320,000 deaths occurred in 2015 globally due to RHD (Watkins et al., 2017). *S. pyogenes* expresses an array of cell wall-associated and secreted virulence factors essential for causing various GAS diseases. Despite the long-standing knowledge of GAS diseases, an effective commercial vaccine against GAS is still unavailable.

In *S. pyogenes*, c-di-AMP regulates cellular activities and virulence factor expression, but the underlying mechanisms are largely unknown. Our previous studies have shown that misregulation of c-di-AMP homeostasis by the deletion of the c-di-AMP synthase gene *dacA* or a phosphodiesterase gene *pde2* leads to the loss of the transcription of the virulence factor SpeB (Fahmi et al., 2019; Faozia et al., 2021). The SpeB null phenotype observed in the Δ*dacA* mutant is mediated via the regulation of the potassium transporter KtrAB (Faozia et al., 2021). However, the mechanism by which the *Δpde2* mutant exhibits the SpeB-null phenotype remains unknown. Through transposon mutagenesis, the first gene in the *dlt* operon, *dltX*, was identified as a suppressor of the SpeB-null phenotype of the *Δpde2* mutant. The primary function of the *dlt* operon is to incorporate D-alanine ester into teichoic acids, resulting in a decreased negative surface charge and increased resistance against the host’s cationic antimicrobial peptides (CAMPs) (Peschel et al., 1999; Kamar et al., 2017).

Given the role of the *dlt* operon in modulating cell surface charge, we also investigated whether or not cell envelope stress influences SpeB production in the Δ*pde2* mutant. Gram-positive bacteria possess a conserved cell envelope stress response regulatory system called LiaFSR, which senses and responds to cell envelope stress induced by CAMPs (Lin et al., 2020). LiaFSR influenced SpeB production in the Δ*pde2* mutant. However, our data revealed that the Dlt and LiaFSR systems independently regulate SpeB production in the Δ*pde2* mutant.

## Results

### The Dlt system impacts the expression of the SpeB virulence factor in the Pde2-deficient mutant of *S. pyogenes*

**Figure 1:**
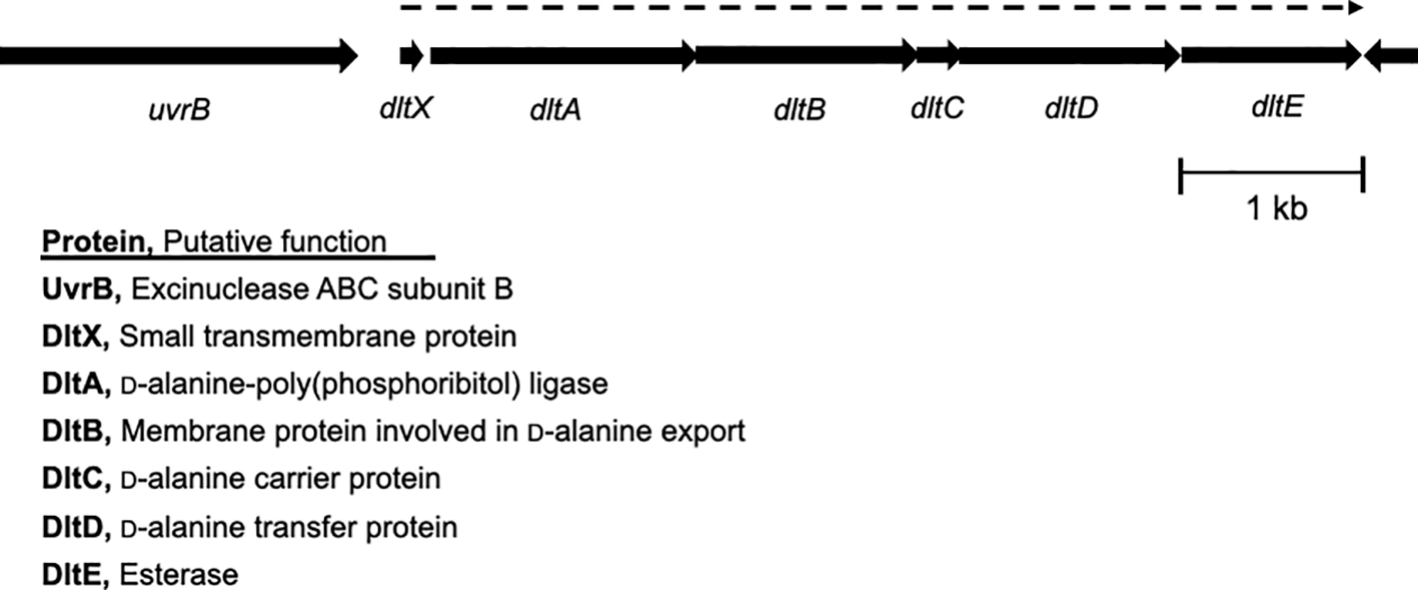
Genetic organization of the *dlt* operon in *S. pyogenes*. The *dlt* operon consists of *dltX, dltA, dltB, dltC, dltD*, and *dltE* genes. Each arrow indicates an individual open reading frame and its orientation. *dltX* is co-expressed with the downstream genes in the *dlt* operon, whose expression is shown with a dotted arrow over the *dlt* genes. The proteins encoded by these open reading frames and their putative functions are shown.

**Figure 2:**
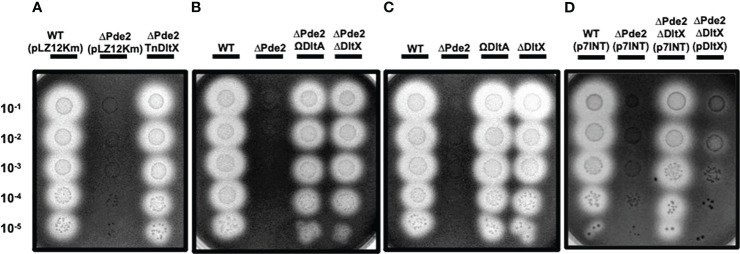
Inactivation of the *dltX* or *dltA* gene restores the SpeB activity of the *Δpde2* mutant. The activity of the secreted protease SpeB is shown on protease indicator plates. Strains were grown overnight and spotted (2µl) onto protease indicator agar plates after serial dilution. SpeB activity displays a clear zone around the spotted cells after incubation. The strains’ names are shown above each image, and the dilution degrees of the spotted cultures are indicated at the left side of the first image. Plates were incubated anaerobically at 37°C for 24 - 48 h. Since pLZ12Km has the same kanamycin resistance gene as the transposon used for the screening, it was used as the control for kanamycin addition to the media. To construct the *dltX*-complemented plasmid, p*dltX*, the p7INT plasmid was used. The following strains were tested: the wild type (WT), *pde2* deletion mutant (ΔPde2), a transposon-generated mutant (*Δ*Pde2TnDltX), *dltX* deletion mutant (ΔDltX), *pde2* and *dltX* double deletion mutant (ΔPde2ΔDltX), *dltA* insertional disruption mutant (ΩDltA), *pde2* deletion and *dltA* insertional disruption mutant (ΔPde2ΩDltA), and *dltX*-complemented Δ*pde2*Δ*dltX* strain (ΔPde2ΔDltX(pDltX). This image is representative of many.

Our previous study demonstrated that deleting a c-di-AMP phosphodiesterase gene, *pde2*, abolished SpeB production at the transcriptional level in *S. pyogenes* (Fahmi et al., 2019). We employed transposon mutagenesis to identify potential genes involved in the regulation of SpeB production in the *Δpde2* mutant. We screened ~3,000 colonies and isolated 24 transposon-generated mutants that showed SpeB activity similar to the wild type. DNA sequencing was performed using primers binding to a transposon sequence to determine the transposon insertion sites of these mutants. This process was successful for 17 mutants. Interestingly, all 17 mutants had a transposon insertion either in the promoter region of or in the *dltX* gene. These mutants were not clones because they all have different transposon insertion sites. The *dlt* operon is highly conserved in Gram-positive bacteria, encoding gene products that incorporate D-alanine ester into teichoic acid molecules (Peschel et al., 1999; Neuhaus and Baddiley, 2003). In *S. pyogenes*, the *dlt* operon comprises six genes, *dltXABCDE.* The first gene, *dltX*, encodes a membrane-associated small protein consisting of 47 amino acids (**Figure 1**). To confirm that *dltX* inactivation is responsible for derepression of the SpeB production in the Δ*pde2* strain, we deleted *dltX* in the Pde2-deficient mutant and evaluated the SpeB activity of this Δ*pde2*Δ*dltX* mutant. Like the transposon-generated mutants, the *dltX* deletion in the Δ*pde2* background restored SpeB activity comparable to that of the wild type (**Figures 2A, B**). To investigate further, we disrupted *dlt*A by inserting a plasmid in the Δ*pde2* background and evaluated its SpeB activity. The resultant Δ*pde2*Ω*dltA* mutant does not express all the genes downstream of *dltA* in the operon due to a polar effect. We chose *dltA* because *dltX* is too small to use this gene disruption method. The strain also produced SpeB at a level equivalent to that of the Δ*pde2*Δ*dltX* mutant (**Figure 2B**). When we added the *dltX* gene back to the Δ*pde2*Δ*dltX*, the SpeB phenotype of the Δ*pde2*Δ*dltX*(p*dltX*) strain was almost the same as that of the Δ*pde2* mutant (**Figure 2D**). These results indicate that the Dlt system affects SpeB production in the Δ*pde2* mutants. The single gene deletion mutants, the Δ*dltX* mutant and Ω*dltA* mutant, showed SpeB activity similar to that of the wild type (**Figure 2C**).

### Deletion of the *dltX* gene increases the negative surface charge of *S. pyogenes*

**Figure 3:**
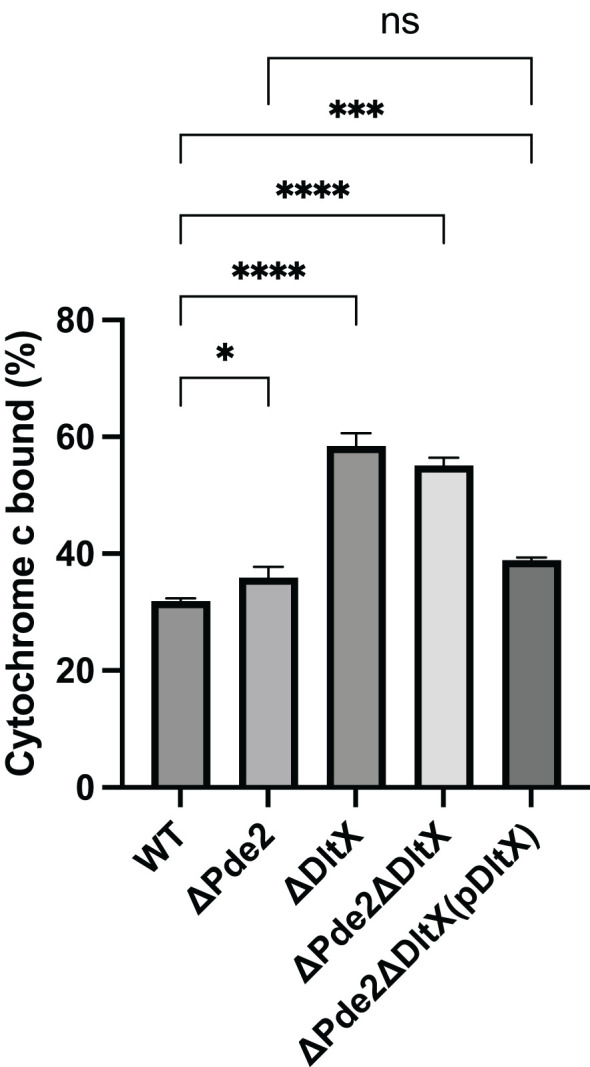
Deletion of *dltX* increases the negative surface charge of *S. pyogenes*. The amount of the cationic protein cytochrome c bound to S*. pyogenes* cell surface was measured to compare the negative surface charge of strains. The data are the means and standard errors of the means derived from three independent experiments. The significance of the difference between two strains was evaluated by a two-tailed unpaired t-test (*, *P* < 0.05; ***, *P* < 0.001; ****, *P* < 0.0001; ns, no significance). The following strains were tested: the wild type (WT), *pde2* deletion mutant (ΔPde2), *dltX* deletion mutant (ΔDltX), *pde2 dltX* double deletion mutant (ΔPde2ΔDltX), *dltX*-complemented *pde2* and *dltX* double deletion mutant [ΔPde2ΔDltX(pDltX)].

Previous research indicates that D-alanylation defects in lipoteichoic acids (LTA) significantly alter the surface charge of GAS (Kristian et al., 2005). In this study, we investigated the role of DltX, a new member of the *dlt* operon in GAS, in modulating cell surface charge. The surface charges of strains were examined using the cationic protein cytochrome c. The *ΔdltX* and *Δpde2ΔdltX* mutants bound more cytochrome c than the wild-type strain (**Figure 3**), indicating that the absence of DltX leads to an increase in negative surface charge. The amount of the cytochrome c bound to the *Δpde2* mutant was a little bit higher than that bound to the wild type, but the difference was much smaller than those between the Δ*dltX* mutants and the wild type (**Figure 3**), suggesting that the absence of Pde2 minimally affects the expression of the *dlt* operon.

### Deleting *dltX* causes increased sensitivity to the cationic antimicrobial peptide polymyxin B

D-alanylation carried out by the Dlt system decreases the affinity of cationic antimicrobial peptides (CAMPs) by reducing the negative surface charge on bacterial cells (Peschel et al., 1999; Kristian et al., 2005). We investigated the effect of *dltX* gene deletion in *S. pyogenes* on the resistance to a CAMP polymyxin B (PMB). *S. pyogenes* strains were cultured with varying concentrations of PMB, and the minimum inhibitory concentrations (MICs) of PMB were measured. The MIC of PMB for both the wild type and Δ*pde2* mutant was 50 µg/ml. In contrast, the MIC for the strains with *dltX* deletion, the Δ*dltX* and Δ*pde2*Δ*dltX* mutants, decreased to 10 µg/ml. These results indicate that the *dltX* deletion in *S. pyogenes* increases the susceptibility of the bacteria to PMB.

### LiaFSR influences SpeB production in the *Δpde2* mutant

**Figure 4:**
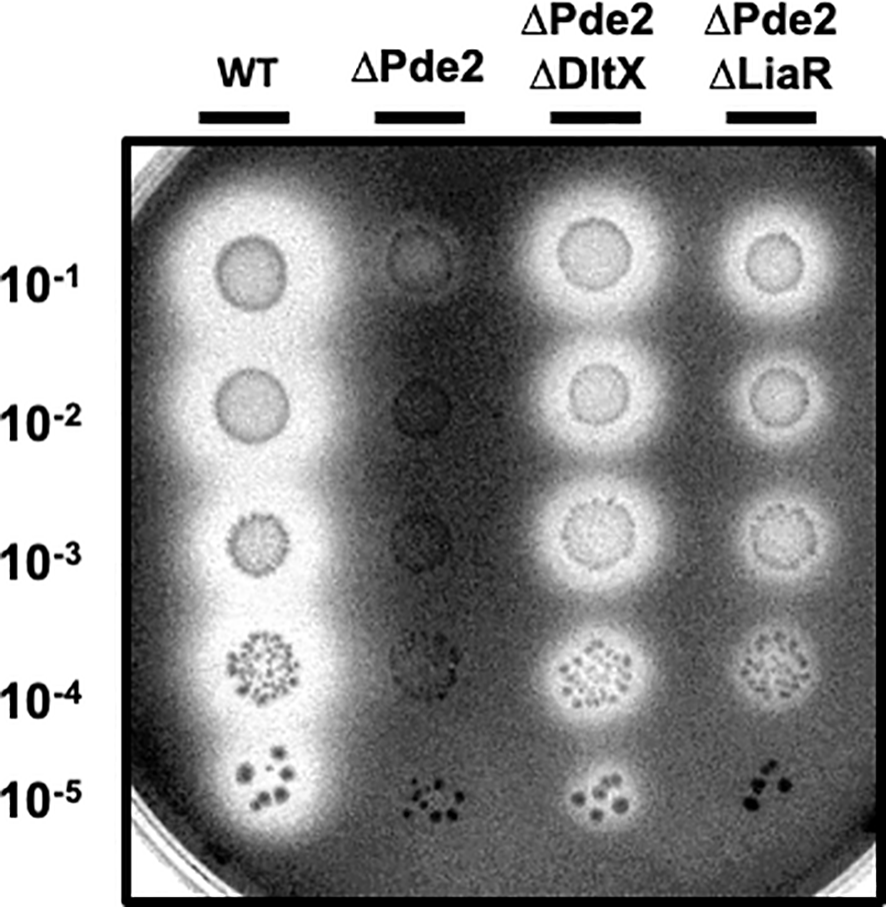
The deletion of *liaR* partially restored the SpeB activity of the *Δpde2* mutant. The clear zones on the protease indicator plates display the activity of SpeB secreted by *S. pyogenes*. The names of the strains used are shown above the image, and the dilution degrees of the overnight cultures are indicated at the left side of the picture. Plates were incubated anaerobically at 37˚C for 24 - 48 h. The following strains were tested: the wild type (WT), *pde2* deletion mutant (ΔPde2), *pde2* and *dltX* double deletion mutant (ΔPde2ΔDltX), and *pde2* and *liaR* double deletion mutant *(Δ*Pde2*Δ*LiaR). This image is representative of many.

The LiaFSR gene regulatory system, which is composed of a membrane-bound repressor protein (LiaF), a sensor kinase (LiaS), and a response regulator (LiaR), functions to detect and responds to cell envelope stress induced by CAMPs (Arias et al., 2011; Lin et al., 2020). Given that the degree of D-alanylation of teichoic acids affects cell envelope charge and both the Dlt and LiaFSR systems respond to CAMPs, we investigated the role of the LiaFSR system in SpeB regulation in the Δ*pde2* mutant. We deleted the response regulator *liaR* in the Δ*pde2* background and measured SpeB activity. The Δ*pde2*Δ*liaR* mutant derepressed SpeB production like the Δ*pde2*Δ*dltX* mutant, although at a lower level (**Figure 4**). This result indicates that LiaFSR also influences SpeB production in the Δ*pde2* mutant.

### The Dlt system and LiaFSR are not linked in the *speB* regulation in the *Δpde2* mutant

**Figure 5:**
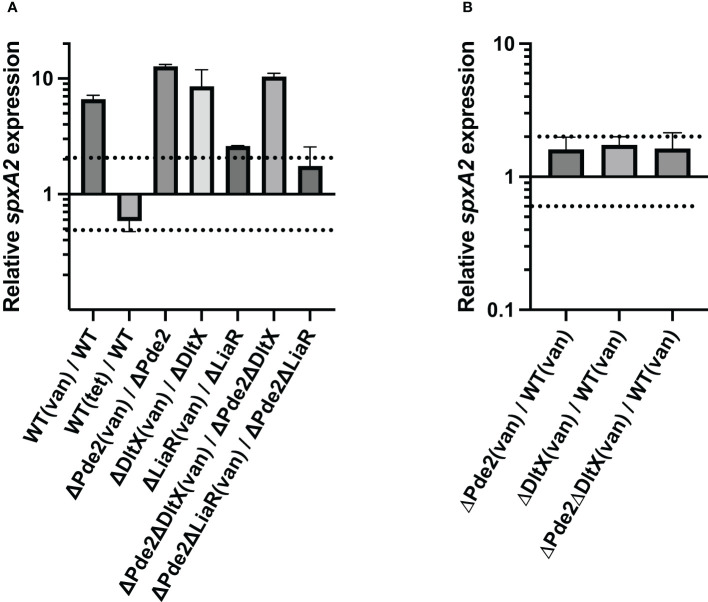
The deletion of *dltX*, *pde2*, or both does not alter *spxA2* expression. The effect of the cell wall stressor vancomycin on *spxA2* expression in *S. pyogenes* was evaluated through real-time qRT-PCR. Cells were grown to the mid-exponential growth phase and harvested for RNA extraction. Data are derived from three independent cultures, and each was assayed in duplicate. The figure shows the means and standard deviations. In the figure, dotted lines represent two-fold difference in transcript levels and serve as a marker for significant differential expression in a quantitative Reverse Transcription Polymerase Chain Reaction (qRT-PCR) assay. The following strains were tested: wild type (WT), *pde2* deletion mutant (ΔPde2), *dltX* deletion mutant (ΔDltX), *dltX* and *pde2* deletion mutant (ΔPde2ΔDltX), *liaR* deletion mutant (ΔLiaR), and *liaR* and *pde2* deletion mutant (ΔPde2ΔLiaR). (van), 0.5 µg/ml vancomycin-treated; (tet), one µg/ml tetracycline-treated.

Since SpeB production was also influenced by LiaFSR in the *Δpde2* mutant (**Figure 4**), and defects in D-alanylation may result in the cell envelope stress by altering cell surface charge, we investigated if SpeB activity restoration by *dlt* mutation in the *Δpde2* background was through the LiaFSR system. LiaFSR responds to cell envelope stressors by regulating the expression of *spxA2* in Gram-positive bacteria (Baker et al., 2020; Sanson et al., 2021). For example, the treatment of the cell wall stressor vancomycin (0.5 µg/ml) stimulates LiaFSR, which enhances the transcription of *spxA2* in *S. pyogenes* (Lin et al., 2020). We measured *spxA2* transcript levels in cells with or without vancomycin treatment (**Figure 5**). As expected, all the strains, except the *liaR* deletion mutants, showed an increased amount of *spxA2* transcript when treated with vancomycin (**Figure 5A**). Moreover, significant changes of the *spxA2* transcript level in the *Δpde2, ΔdltX*, and *Δpde2ΔdltX* mutants compared to that of the wild type was not observed when they were treated with vancomycin (**Figure 5B**). A negative control, tetracycline (1 µg/ml), a protein synthesis inhibitor, showed no significant change (less than twofold) of the *spxA2* transcript level. These results suggest that neither c-di-AMP levels in cells nor D-alanylation of teichoic acids affect the expression of *spxA2.*

### The deletion of *dltX*, not *liaR*, in the Δ*pde2* mutant increases the level of cellular c-di-AMP

**Figure 6:**
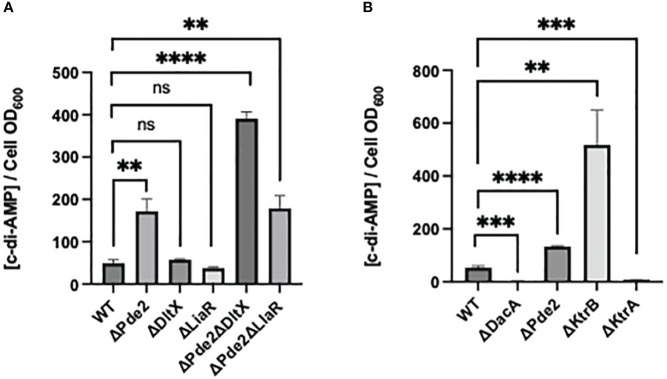
c-di-AMP concentration in the cell extracts of *S. pyogenes* strains. The c-di-AMP amount (pmol/cell OD_600_) in each strain in the exponential phase was measured using a competitive ELISA. The significance of the difference between each pair of strains was evaluated by two-tailed unpaired t-tests (**, *P* < 0.01; ***, *P* < 0.001; ****, *P* < 0.0001; ns, no significance). The following strains were tested: wild type (WT), *pde2* deletion mutant (ΔPde2), *dltX* deletion mutant (ΔDltX), *liaR* deletion mutant (ΔLiaR), *pde2* and *dltX* deletion mutant (ΔPde2ΔDltX), *pde2* and *liaR* deletion mutant (ΔPde2ΔLiaR), *dacA* deletion mutant (ΔDacA), *ktrB* deletion mutant (ΔKtrB) and *ktrA* deletion mutant (ΔKtrA).

The amounts of c-di-AMP levels of strains were measured. Both the *dltX* and *liaR* deletion in the wild type did not change cellular c-di-AMP level, but all *pde2* deletion mutants, the Δ*pde2*, Δ*pde2*Δ*dltX*, and Δ*pde2*Δ*liaR* mutants produced increased amounts of c-di-AMP (**Figure 6A**). These increases agree to previous studies since the deletion of a c-di-AMP phosphodiesterase gene, *pde2* or *gdpP* increases cellular c-di-AMP level in *S. pyogenes* (Fahmi et al., 2019). Interestingly, *dltX* deletion in the Δ*pde2* mutant increased c-di-AMP amount further, but *liaR* deletion in the Δ*pde2* mutant did not affect c-di-AMP production.

### K^+^ transport capacity and cellular c-di-AMP level show an inverse relationship in *S. pyogenes*

We investigated if the impaired capability of K^+^ transport affects cellular c-di-AMP levels. The Δ*ktrB* strain, the mutant with the deletion of the high-affinity K^+^ transporter gene, produced a high amount of c-di-AMP, even more than that of the Δ*pde2* mutant (**Figure 6B**). The Δ*ktrA* strain, the mutant with the deletion of K^+^ transport inhibitor gene, produced a lower amount of c-di-AMP level than that of the wild type. These results indicate that the relationship between K^+^ transport capability and c-di-AMP production is inversely proportional in *S. pyogenes*.

**Figure 7:**
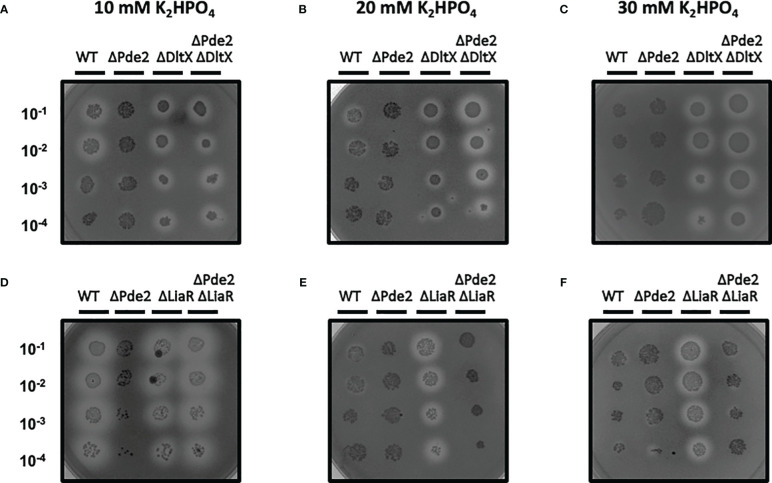
The SpeB activity phenotype of ΔPde2ΔDltX did not change at a high salt condition, unlike ΔPde2ΔLiaR. The activity of the secreted protease SpeB is shown on protease indicator plates. Strains were grown overnight and spotted (2µl) onto protease indicator agar plates after serial dilution. SpeB activity displays a clear zone around the spotted cells after incubation. The strains’ names are shown above each image, and the dilution degrees of the spotted cultures are indicated at the left side of the first image. Plates were incubated anaerobically at 37°C for 24 - 48 h. The following strains were tested: the wild type (WT), *pde2* deletion mutant (ΔPde2), *dltX* deletion mutant (ΔDltX), *pde2* and *dltX* deletion mutant (ΔPde2ΔDltX), *liaR* deletion mutant *(Δ*LiaR), and *pde2* and *liaR* deletion mutant *(Δ*Pde2*Δ*LiaR). This image is representative of many.

The SpeB phenotype of the Δ*pde2*Δ*dltX* mutant was not changed in high salt media, unlike the wild type and Δ*pde2*Δ*liaR* mutant. We examined the SpeB activities of strains in high-salt media. Regular C medium used to make protease detection plates contains 10 mM K_2_HPO_4_. To increase salt concentration, we doubled or tripled the amount of K_2_HPO_4_. In these higher salt C media, the SpeB activities of the wild type and Δ*pde2*Δ*liaR* mutants were reduced (**Figure 7**). However, the SpeB activities of the Δ*pde2*Δ*dltX* mutant did not change in higher salt media. Thus, DltX and LiaR respond differently to a high osmolarity condition in the Δ*pde2* background. The Δ*dltX* and Δ*liaR* mutants still showed SpeB activity in high salt conditions.

## Discussion

In a previous study, we observed that the transcription of the virulence factor gene *speB* ceases when the c-di-AMP phosphodiesterase *pde2* gene is deleted in *S. pyogenes* (Fahmi et al., 2019). In this current research, we investigated the regulatory mechanisms and found that the Dlt system and LiaFSR regulate SpeB production through different mechanisms in the Δ*pde2* mutant.

The *dlt* operon, found in nearly all Gram-positive bacteria, is comprised mostly of four to five genes - *dlt(X)ABCD*. This operon is primarily responsible for incorporating D-alanine esters into teichoic acids. In previous studies, this D-alanylation is entirely abolished when any of the *dlt* core genes (*dltA* to *dltD*) is inactivated in bacteria, including *S. aureus, S. pyogenes, S. pneumoniae, Enterococcus faecalis*, and Group B *Streptococcus* (Peschel et al., 1999; Boyd et al., 2000; Abachin et al., 2002; Kristian et al., 2005; Koprivnjak et al., 2006; Kovács et al., 2006). Recently, *dltX* has been discovered as an essential gene for D-alanylation in *B. thuringiensis*, but the specific role of the small membrane-associated protein DltX in this process remains unstudied (Kamar et al., 2017). Our study also suggests that DltX is essential for D-alanylation in *S. pyogenes* because the *dltX* deletion strain showed the same phenotype as the strain with a core *dlt* gene disruption. The mutation in the *dlt* operon in bacteria leads pleiotropic effects, including increased negative cell surface charge, increased sensitivity to CAMPs, enhanced autolysis, increased acid sensitivity, decreased biofilm formation, reduced adhesion to epithelial cells, and expression of altered virulence properties (Peschel et al., 1999; Boyd et al., 2000; Abachin et al., 2002; Kristian et al., 2005; Koprivnjak et al., 2006; Kovács et al., 2006).

D-alanylation of teichoic acid is a crucial bacterial defense mechanism against cationic antimicrobial peptides (CAMPs) (Kamar et al., 2017). The gram-positive thick cell wall plays a significant role in shielding against the host’s antimicrobial peptides (AMPs). More than 60% of the total mass of the cell wall is made up of negatively charged teichoic acids (TAs) (Neuhaus and Baddiley, 2003; Silhavy et al., 2010). TAs are commonly found as wall teichoic acids (WTAs) or lipoteichoic acids (LTAs). WTAs are linked to peptidoglycan, and LTAs are anchored to the cytoplasmic membrane via their glycolipid moiety (Fischer, 1988; Fischer et al., 1990). The critical components of TAs are disaccharide anchors and phosphodiester-linked polyglycerol phosphate or polyribitol phosphate, which contributes to its net negative surface charge (Kojima et al., 1985; Bera et al., 2007). The positively charged AMPs (e.g., cathelicidins, defensin, etc.) are electrostatically attracted by the negatively charged bacterial surface and damage the bacterial cell membrane. Bacteria can modify their cell surface charge by incorporating positively charged residues to counteract CAMPs. This modification can be carried out by adding L-lysine to phosphatidylglycerol mediated by the *mprF* gene or incorporating D-alanine ester on free hydroxyls of the repeating sugar mediated by *dlt* operon as has been observed in firmicutes (Peschel et al., 1999; Abachin et al., 2002; Kristian et al., 2005; Weidenmaier and Peschel, 2008; Cox et al., 2009; Saar-Dover et al., 2012). In *Streptococcus*, D-alanylation appears to be the primary mechanism of CAMP resistance. D-alanine incorporation most likely modifies the conformation of LTAs, increasing cell wall density and decreasing CAMP penetration (Saar-Dover et al., 2012).

In *S. pyogenes*, the *dlt* operon consists of six genes *dltXABCDE*. DltX is a small protein with 47 amino acids. The absence of the *dltA* gene, essential for D-alanylation, significantly lowers the expression of GAS virulence factors, including M protein and SIC (streptococcal inhibitor of complement) protein (Cox et al., 2009). *S. pyogenes* cell envelope TAs are mostly lipoteichoic acids. The loss of lipoteichoic acid D-alanylation by *dltA* mutation displays an increased negative surface charge, enhanced susceptibility to AMP, lysozyme, and neutrophil killing, in addition to decreased adhesion and invasion into the human pharyngeal epithelial cells (Kristian et al., 2005). Our findings demonstrate that along with c-di-AMP, the Dlt system influences the production of the virulence factor SpeB in *S. pyogenes*. Inactivation of *dltA* or *dltX* in the *Δpde2* background restores SpeB production to the wild-type level (**Figure 2B**). Also, this study shows that the *ΔdltX* mutant displays increased negative surface charge (**Figure 3**) and is more susceptible to cationic antimicrobial peptide polymyxin B than the wild type. These findings suggest that the *dltX* gene deletion causes D-alanylation defects, which increase negative surface charge and are more attracted to cationic polymyxin B. Similar results were observed in *B. thuringiensis*, where *dltX* is essential for D-alanylation (Kamar et al., 2017). The SpeB derepression by *dlt* mutation in the *Δpde2* mutant appears not to be linked to the net negative surface charge. Even though the loss of the *pde2* gene drastically reduces *speB* expression, the surface charge of the *Δpde2* mutant was not changed (**Figure 3**). This could suggest that the deletion of the *pde2* gene does not affect D-alanylation of teichoic acid. As expected, the *Δpde2ΔdltX* mutant with restored SpeB production bound almost twice more cationic cytochrome c than the amount bound to the wild type, probably because of the lack of D-alanylation (**Figure 3**).

Since D-alanylation of teichoic acid alters the cell surface charge, it may influence cell envelope stress in Gram-positive bacteria. Prior work has shown that the *dlt* mutation in *B. subtilis* affects the LiaFSR three-component system that can sense and respond to cell envelope stress. The Δ*dltD* mutation in *B. subtilis* increased LiaFSR activity, most likely because of its cell membrane stress induced by increased surface negative charge (Mascher, 2006; Hyyryläinen et al., 2007; Lin et al., 2020). In *S. pyogenes*, the sensor kinase LiaS and the repressor protein LiaF are colocalized in the cell membrane. They are involved in microdomain (Exportal) formation, and the disruption of GAS Exportal by CAMPs activates the LiaFSR system (Lin et al., 2020). Mutation in LiaFSR alters SpeB activity; Δ*liaR* mutation significantly increases *speB* transcription through SpxA2 (Sanson et al., 2021). In this study, Δ*liaR* in the *Δpde2* mutant derepressed SpeB production, but the derepression degree was less than that of Δ*dltX* (**Figure 4**).

The RNA polymerase binding protein SpxA is a transcriptional regulator commonly found in firmicutes (Port et al., 2017; Lin et al., 2020). Several gram-positive bacteria have two highly similar SpxA paralogs, SpxA1 and SpxA2 (Nakano et al., 2005; Port et al., 2017). SpxA1 mainly responds to oxidative stress, and SpxA2 regulates numerous cellular activities such as cell division, cell wall homeostasis, fatty acid biosynthesis, virulence regulation, biofilm formation, as well as cell envelope stress regulation (Kajfasz et al., 2010; Baker et al., 2014; Kajfasz et al., 2015; Baker et al., 2020). SpxA1 and SpxA2 exist in *S. pyogenes* and have an opposite effect on virulence regulation or stress response (Port et al., 2017). The Δ*spxA1* mutant is highly attenuated and shows enhanced PMB resistance, while Δ*spxA2* becomes hypervirulent and more sensitive to PMB (Port et al., 2017). SpxA2 negatively regulates *speB* expression (Port et al., 2017). GAS *spxA2* transcription is highly dependent on the LiaFSR system, similar to other firmicutes (Nakano et al., 2003; Baker et al., 2020; Sanson et al., 2021). Our qRT–PCR data confirmed that LiaR is essential for the *spxA2* gene expression (**Figure 5**). The *spxA2* transcript level was significantly reduced in the *ΔliaR* mutants compared to the wild type or the *Δpde2* mutant in the presence of the cell wall stressor vancomycin (**Figure 5**). The transcription of *spxA2* did not change in the *Δpde2, ΔdltX*, or *Δpde2ΔdltX* mutant compared to the wild type in the presence of vancomycin (**Figure 5**). These findings indicate that SpeB restoration of the *Δpde* mutant by *dlt* mutation is not through the LiaFSR-regulated gene *spxA2*. We observed no significant change in the *liaR* transcript level in the *Δpde2* or *ΔdltX* mutant compared to that of the wild type or the *Δpde2* mutant. Also, the transcription of *dltX* was not altered in the *Δpde2* or *ΔliaR* mutant relative to the wild type or the *Δpde2* mutant (Unpublished data). Thus, the Dlt system and LiaFSR are probably not linked in regulating SpeB production in the *Δpde2* mutant.

It was previously shown that cellular c-di-AMP level controls K^+^ transporter activity in *S. pyogenes* (Faozia et al., 2021). This study examined whether or not K^+^ transport capability change affects cellular c-di-AMP levels. When the high-affinity transporter gene *ktrB* was deleted, cells produced a high amount of c-di-AMP. However, less c-di-AMP was generated when the transporter regulator (inhibitor) gene *ktrA* was deleted. Thus, K^+^ transport capacity has an inverse relationship to cellular c-di-AMP level in *S. pyogenes*. Cellular c-di-AMP level is changed by environmental osmolarity alteration in several gram-positive bacteria, including *Lactococcus lactis, Lactobacillus plantarum, Listeria monocytogenes*, and *S. aureus* (Pham et al., 2018). When the bacteria encounter high osmolarity conditions, cellular c-di-AMP level decreases rapidly. Under a low osmolarity condition, cellular c-di-AMP levels in these bacteria increase (Pham et al., 2018). Thus, these results, including ours, demonstrate that bacteria alter cellular c-di-AMP levels based on environmental or cellular conditions and change cellular activities based on c-di-AMP levels. However, the molecular mechanism of how these bacteria sense environmental or cellular conditions to change c-di-AMP level has yet to be elucidated.

The Δ*pde2*Δ*dltX* mutant produces a higher amount of c-di-AMP than that of the Δ*pde2* or Δ*pde2*Δ*liaR* mutant (**Figure 6**). Since cellular c-di-AMP level can be altered by osmolarity or turgor pressure change (Pham et al., 2018), we examined the SpeB phenotypes of strains in different salt concentration conditions. The single gene deletion mutants, the Δ*dltX* mutant and Δ*liaR* mutant, behaved similarly; they showed identical SpeB phenotypes regardless of salt concentration change. However, the Δ*pde2*Δ*dltX* mutant and Δ*pde2*Δ*liaR* mutant behaved differently. The Δ*pde2*Δ*liaR* mutant showed reduced SpeB production, but the SpeB production of the Δ*pde2*Δ*dltX* mutant did not decrease in higher salt media. This indicates that the DltX and LiaFSR systems affect SpeB production through different pathways in the Δ*pde2* background.

In summary, our results show that both the Dlt and LiaFSR systems affect SpeB production in the Δ*pde2* background. The LiaFSR system controls *speB* expression through the transcriptional regulator SpxA2. We could not pinpoint the regulatory pathway the Dlt system involves, but salt transport or turgor pressure regulation might be involved. We also proved that the small membrane protein DltX is essential for the D-alanylation of teichoic acids in *S. pyogenes*.

## Materials and methods

### Bacterial strains and media

*S. pyogenes* HSC5 (*emm* genotype 14) (Hanski et al., 1992; Port et al., 2013) was employed for all experiments, including strain construction. Molecular cloning experiments utilized *Escherichia coli* DH5α or TOP10 (Invitrogen), which was cultured in Luria-Bertani broth. The routine culture of *S. pyogenes* employed Todd-Hewitt medium (BBL) supplemented with 0.2% yeast extract (Difco) (THY medium), and cells were grown at 37°C in sealed tubes without agitation. Unless otherwise indicated, C medium (Lyon et al., 1998) was used to grow *S. pyogenes* for SpeB activity assay and RNA preparation for real-time qRT-PCR. Bacto agar (1.4%, w/v; Difco) was added to make solid media. Cultures on solid media were incubated under the anaerobic condition created by a commercial product (e.g., GasPak; catalog no. 260678; BBL). When appropriate, antibiotics were added to the media at the following concentrations if they are not specified: kanamycin, 50µg/ml for *E. coli* and 500µg/ml for *S. pyogenes*; erythromycin, 500µg/ml for *E. coli* and one µg/ml for *S. pyogenes*.

### Manipulation of DNA

Plasmid DNA was isolated via a commercial kit (e.g., Gene Elute plasmid miniprep kit; Sigma) and used to transform *S. pyogenes* or *E. coli* as described previously (Caparon et al., 1991). Enzymes for DNA cloning and PCR were used according to the manufacturers’ recommendations. Chromosomal DNA was purified from *S. pyogenes* using a commercial kit (e.g., GenElute bacterial genomic DNA kit; Sigma).

### Transposon mutagenesis

For the transposon mutagenesis, *Tn*ΩKm2, a *Tn*4001 derivative containing a kanamycin resistance determinant, was employed (Kang et al., 2012). Briefly, the purified plasmid containing *Tn*ΩKm2 was introduced to the Δ*pde2* mutant using electroporation (voltage: 2100V, Capacitor: 25 uF, Resistance: 200 Ohms). The colonies with kanamycin resistance were patched on the protease indicator plates, and the strains showing the wild-type level protease activity were selected. The transposon insertion sites in those strains were identified by sequencing of chromosomal DNA with a primer binding to a transposon sequence. The sequencing data were compared with the NCBI genomic database (http://www.ncbi.nlm.nih.gov/BLAST/) to identify transposon-insertion sites.

### Strain construction

The generation of the *pde2* deletion mutant has been described elsewhere (Fahmi et al., 2019). Other gene deletion mutations, Δ*dltX* and Δ*liaR* on chromosomal loci were generated by employing the shuttle vector with a temperature-sensitive replication origin, pJRS233 (Cho and Kang, 2013). Briefly, the target gene and ~ 1000 bp sequences immediately upstream and downstream were amplified by PCR. This PCR product was inserted into pJRS233 using the fast-cloning method (Li et al., 2011). The plasmid with a target gene deletion allele was obtained by inverse PCR. The target gene deletion plasmids, pΔ*dltX* and pΔ*liaR*, were created using this method. The primers used to generate the PCR products are listed in **Table 1**. pΔ*dltX* or pΔ*liaR* was used to replace each target gene in the wild type or the Δ*pde2* mutant by the gene deletion method that employs the temperature-sensitive replication origin, as described previously (Cho and Kang, 2013). The Ω*dltA* or Δ*pde2ΩdltA* mutant was constructed by insertional disruption of the *dltA* gene through single homologous recombination. An internal region of the *dltA* gene was amplified by PCR and then inserted into the suicide vector pCIV2 (Okada et al., 1993; Lyon et al., 2001). The resultant plasmid, pΩ*dltA*, was used to transform HSC5 or the Δ*pde2* mutant into resistance to kanamycin. Since the suicide vector pCIV2 lacks the replication origin of *S. pyogenes*, the plasmid can only exist in *S. pyogenes* by integrating into the chromosome through homologous recombination. The fidelity of all genetic constructs was confirmed by PCR and/or DNA sequencing.

**Table 1:** Primers used.

Name	Sequence^a^	Remarks
Mutagenic Primers^b^		
To create pΔ*dltX*		
FC5p7INT-2 FC3p7INT-2	cctgtgtgaaattgttatccgctc gtcgtgactgggaaaaccctgg	For vector amplification (4091 bps)^d^
5*dltX*1000 3*dltX*1000	gggttttcccagtcacgacCGACTGGGCTACTTGATCCTGG gcggataacaatttcacacaggGAATCTGGTTTGGGGTAGCCAA	For insert amplification (2185 bps)^d^
To create p*dltX*		
FC5p7INT-2 FC3p7INT-2	cctgtgtgaaattgttatccgctc gtcgtgactgggaaaaccctgg	For vector amplification (4178 bps)^d^
5dltX-p7INT 3dltX-p7INT	ggttttcccagtcacgacCTGAAGGAAGATCTGGATCC cggataacaatttcacacaggGGCTCTCTTGGTCGTCAGAC	For insert amplification (763 bps)^d^
To create pΩ*dltA*		
5pUC18 3pUC18	cgggtaccgagctcgaattcg cctgcaggcatgcaagcttg	For vector amplification (4228 bps)^d^
5KO*dltA* 3KO*dltA*	cttgcatgcctgcaggCCTTGTCTCACTATCAGAGATTGAGTCAG cgagctcggtacccgCACCCTGCTCCCCTGA	For insert amplification (692 bps)^d^
To create pΔ*liaR*		
FC5p7INT-2 FC3p7INT-2	cctgtgtgaaattgttatccgctc gtcgtgactgggaaaaccctgg	For vector amplification (4091 bps)^d^
5*liaR*1000 3*liaR*1000	gggttttcccagtcacgacGGATAGGCGATGAAAAAACGTTACTATGC gcggataacaatttcacacaggCATCATAGTACCCTTCTTTAGCCAAACC	For insert amplification (2691 bps)^d^
Analysis primers^c^		
RT*spxA1*-F RT*spxA1*-R	ACAAGTCCATTAAGCCGTGATG AGGGCGACGAAGAAGACTTG	
RT*spxA2*-F RT*spxA2*-R	GAACTTAGGAAAAAGAACCGCTAACTAA CGCAATCGAGAGCTTTGGC	
RT*dltX*-F RT*dltX*-R	TCAAGAATGAGAGGAATTGCTG ACCAAAGAAATAGACCAGCAAC	
RT*liaR*-F RT*liaR*-R	CGTGAAGGGGTTGATTTGGC TAACCCTTCGCTCCTGCATC	
RT*gyrA*-F RT*gyrA*-R	AACAACTCAAACAGGTCGGG CTCCTTCACGGCTAGATTC	

a Sequences are shown 5′ to 3′. Uppercase sequences anneal to the HSC5 chromosome, and lowercase sequences anneal to plasmid sequences.

b Mutagenesis primers were used for PCR reactions to amplify DNA segments used to construct plasmids for gene deletion or insertional gene disruption.

c Analysis primers were used in real-time qRT-PCR to measure the level of gene transcription.

d PCR product size.

The *dltX*-complemented Δ*pde2*Δ*dltX*(p*dltX*) strain was generated using the plasmid p7INT that inserts into the streptococcal chromosome (McShan et al., 1998). The DNA containing the *dltX* gene and its promoter region was amplified by PCR and inserted into p7INT through the fast-cloning method (Li et al., 2011). The resulting plasmid p*dltX* was transferred into the Δ*pde2*Δ*dltX* mutant to make the Δ*pde2*Δ*dltX*(p*dltX*) strain.

### Determination of MIC to polymyxin B

The susceptibility of mutant strains to the cell membrane-targeting antibiotic polymyxin B was monitored as follows. *S. pyogenes* cells grown in THY medium overnight were inoculated into fresh THY medium containing polymyxin B (100, 50, 10, 5, 1, 0.5, and 0 µg/ml). Cells were then grown in a 96-well plate overnight at 37°C, and the OD_600_ of the overnight cultures (~18 h post-inoculation) was measured to determine the final cell density. This experiment was performed in triplicate.

### Cytochrome c binding assay

THY media (50 ml) were inoculated with 2-3% overnight cultures and cultivated to the early exponential phase. Cells were collected by centrifugation at 7000 g for 10 min, resuspended in 50 ml of chemically defined media (DMEM), and incubated overnight. Then, the cells were washed twice with 20 ml of morpholino propanesulfonic acid (MOPS) buffer (20 mM, pH 7), adjusted to the final cell OD_600_ to 3 in 2 ml MOPS buffer with 0.2 mg/ml cytochrome c (Sigma-Aldrich, St. Louis, MO), and incubated for 10 minutes in a shaker at room temperature. As a control, 0.2 mg/ml cytochrome c was incubated in MOPS buffer under the same conditions without bacteria. After 10 min, cells were removed by centrifugation, and the cytochrome c content of the supernatants was quantified photometrically by measuring OD_530_.

### qRT-PCR

Real-time qRT-PCR was conducted as described elsewhere (Cho and Kang, 2013). The primers for qRT-PCR are listed in **Table 1**. The gyrase A subunit gene (*gyrA*) was used as the internal reference gene to normalize the expression level of a specific transcript between samples (Kang et al., 2010). The reported data represent the means and standard errors from three independent assays performed on different days with new RNA samples.

### Determination of gene expression under the influence of an antibiotic

Cells at the exponential phase (0.3 – 0.4 of OD_600_) were collected and incubated in a fresh medium with an antibiotic at 37°C for an hour. Then, cells were collected and lysed for RNA purification.

### SpeB activity measurement using protease indicator plates

Overnight cultures in THY medium were serially diluted with fresh THY medium, and the diluted cells (2µl) were spotted onto protease indicator agar plates (C medium agar plates containing 2% skim milk). The plates were then incubated anaerobically at 37°C for 24 h to 48 h, and SpeB activity that displays a clear zone around the spotted cells was observed.

### Quantification of c-di-AMP in cell extracts by ELISA

The measurement of c-di-AMP concentration in cell extract was conducted as previously described (Fahmi et al., 2019). Briefly, *S. pyogenes* strains were grown to the exponential phase (OD600 = ~0.4) in 10 ml THY medium, washed three times with PBS, resuspended in 1 ml PBS, and lysed through PlyC treatment. The clear supernatant of the culture was collected in a fresh tube after centrifugation at 7,000 relative centrifugal force (rcf) at 4°C for 10 min. The cell lysates were then boiled for 10 min. Clear supernatants were collected after centrifugation and stored at -20°C until used to measure c-di-AMP concentration. The purified CabP protein was diluted to 50 µg/ml in coating buffer (50 mM Na2CO3, 50 mM NaHCO3, pH 9.6), and 100 µl of the solution was added to each well to coat the wells of a 96-well flat-bottom plate. The plates were sealed with plastic wrap and incubated overnight at 4°C. The coated wells were then washed three times with PBS containing 0.05% Tween 20 (PBST) and blocked with 5% bovine serum albumin (BSA). The cell extract samples were diluted (5 times) with 50 mM Tris buffer (pH 8). 100µl of controls, standards, and samples were added to the coated wells (in triplicate). The plates were incubated for 2 hrs at room temperature. Each well of the plates was washed three times with 200 µl PBST. Next, 100 µl of 0.1 µg/ml high-performance streptavidin (Thermo Fisher Scientific) in PBS was added and incubated for 1 hr. After wells were washed three times with PBST, 100 µl of the substrate (0.5 mg of o-phenylenediamine dihydrochloride [Sigma-Aldrich] in citrate buffer [pH 5] containing 20 µl H_2_O_2_) was added to each well and incubated for 30 min at room temperature. Finally, the reactions were stopped with 100 µl of 2M H_2_SO_4_. The OD_492_ of each well was measured using a plate reader. A standard curve was generated and used to measure the levels of c-di-AMP in samples.

### Statistical testing

Each statistical test applied to the experiments was described in the figure legends.

## Data availability statement

The original contributions presented in the study are included in the article/supplementary material. Further inquiries can be directed to the corresponding author.

## Author contributions

SF: Formal Analysis, Investigation, Methodology, Writing – original draft. TH: Investigation, Methodology, Writing – review & editing. KC: Conceptualization, Formal Analysis, Funding acquisition, Investigation, Resources, Supervision, Validation, Visualization, Writing – original draft, Writing – review & editing.

## References

[B1] Abachin E. Poyart C. Pellegrini E. Milohanic E. Fiedler F. Berche P.. (2002). Formation of D-alanyl-lipoteichoic acid is required for adhesion and virulence of *Listeria monocytogenes* . Mol. Microbiol. 43, 1–14. doi: 10.1046/j.1365-2958.2002.02723.x 11849532

[B2] Andrade W. A. Firon A. Schmidt T. Hornung V. Fitzgerald K. A. Kurt-Jones E. A.. (2016). Group B streptococcus degrades cyclic-di-AMP to modulate STING-dependent type I interferon production. Cell Host Microbe 20, 49–59. doi: 10.1016/j.chom.2016.06.003 27414497 PMC5382021

[B3] Arias C. A. Panesso D. Mcgrath D. M. Qin X. Mojica M. F. Miller C.. (2011). Genetic basis for in *vivo* daptomycin resistance in enterococci. N. Engl. J. Med. 365, 892–900. doi: 10.1056/NEJMoa1011138 21899450 PMC3205971

[B4] Baker J. L. Derr A. M. Karuppaiah K. Macgilvray M. E. Kajfasz J. K. Faustoferri R. C.. (2014). *Streptococcus mutans* NADH oxidase lies at the intersection of overlapping regulons controlled by oxygen and NAD+ levels. J. Bacteriol. 196, 2166–2177. doi: 10.1128/JB.01542-14 24682329 PMC4054193

[B5] Baker J. L. Saputo S. Faustoferri R. C. Quivey R. G. Jr. (2020). *Streptococcus mutans* SpxA2 relays the signal of cell envelope stress from LiaR to effectors that maintain cell wall and membrane homeostasis. Mol. Oral. Microbiol. 35, 118–128. doi: 10.1111/omi.12282 32043713 PMC7202993

[B6] Barker J. R. Koestler B. J. Carpenter V. K. Burdette D. L. Waters C. M. Vance R. E.. (2013). STING-dependent recognition of cyclic di-AMP mediates type I interferon responses during *Chlamydia trachomatis* infection. mBio 4, e00018-00013. doi: 10.1128/mBio.00018-13 23631912 PMC3663186

[B7] Bera A. Biswas R. Herbert S. Kulauzovic E. Weidenmaier C. Peschel A.. (2007). Influence of wall teichoic acid on lysozyme resistance in *Staphylococcus aureus* . J. Bacteriol. 189, 280–283. doi: 10.1128/JB.01221-06 17085565 PMC1797201

[B8] Boyd D. A. Cvitkovitch D. G. Bleiweis A. S. Kiriukhin M. Y. Debabov D. V. Neuhaus F. C.. (2000). Defects in D-alanyl-lipoteichoic acid synthesis in *Streptococcus mutans* results in acid sensitivity. J. Bacteriol. 182, 6055–6065. doi: 10.1128/JB.182.21.6055-6065.2000 11029425 PMC94739

[B9] Caparon M. G. Stephens D. S. Olsen A. Scott J. R. (1991). Role of M protein in adherence of group A streptococci. Infect. Immun. 59, 1811–1817. doi: 10.1128/iai.59.5.1811-1817.1991 2019444 PMC257920

[B10] Carapetis J. R. Steer A. C. Mulholland E. K. Weber M. (2005). The global burden of group A streptococcal diseases. Lancet Infect. Dis. 5, 685–694. doi: 10.1016/S1473-3099(05)70267-X 16253886

[B11] Cho K. H. Kang S. O. (2013). *Streptococcus pyogenes* c-di-AMP phosphodiesterase, GdpP, influences SpeB processing and virulence. PloS One 8, e69425. doi: 10.1371/journal.pone.0069425 23869242 PMC3711813

[B12] Commichau F. M. Heidemann J. L. Ficner R. Stülke J. (2019). Making and breaking of an essential poison: the cyclases and phosphodiesterases that produce and degrade the essential second messenger cyclic di-AMP in bacteria. J. Bacteriol. 201. doi: 10.1128/JB.00462-18 PMC6287462 30224435

[B13] Corrigan R. M. Abbott J. C. Burhenne H. Kaever V. Gründling A. (2011). c-di-AMP is a new second messenger in *Staphylococcus aureus* with a role in controlling cell size and envelope stress. PloS Pathog. 7, e1002217. doi: 10.1371/journal.ppat.1002217 21909268 PMC3164647

[B14] Corrigan R. M. Gründling A. (2013). Cyclic di-AMP: another second messenger enters the fray. Nat. Rev. Microbiol. 11, 513–524. doi: 10.1038/nrmicro3069 23812326

[B15] Cox K. H. Ruiz-Bustos E. Courtney H. S. Dale J. B. Pence M. A. Nizet V.. (2009). Inactivation of DltA modulates virulence factor expression in Streptococcus pyogenes. PloS One 4, e5366. doi: 10.1371/journal.pone.0005366 19401780 PMC2671602

[B16] Fahmi T. Faozia S. Port G. C. Cho K. H. (2019). The second messenger c-di-AMP regulates diverse cellular pathways involved in stress response, biofilm formation, cell wall homeostasis, SpeB expression, and virulence in *Streptococcus pyogenes* . Infect. Immun. 87, e00147-00119. doi: 10.1128/IAI.00147-19 30936159 PMC6529668

[B17] Faozia S. Fahmi T. Port G. C. Cho K. H. (2021). c-di-AMP-regulated K+ Importer ktrAB affects biofilm formation, stress response, and speB expression in *Streptococcus pyogenes* . Infect. Immun. 89. doi: 10.1128/IAI.00317-20 PMC8090949 33468578

[B18] Fischer W. (1988). Physiology of lipoteichoic acids in bacteria. Adv. Microbial Physiol. 29, 233–302. doi: 10.1016/S0065-2911(08)60349-5 3289326

[B19] Fischer W. Mannsfeld T. Hagen G. (1990). On the basic structure of poly (glycerophosphate) lipoteichoic acids. Biochem. Cell Biol. 68, 33–43. doi: 10.1139/o90-005 2350496

[B20] Gándara C. Alonso J. C. (2015). DisA and c-di-AMP act at the intersection between DNA-damage response and stress homeostasis in exponentially growing *Bacillus subtilis* cells. DNA Repair (Amst) 27, 1–8. doi: 10.1016/j.dnarep.2014.12.007 25616256

[B21] Hanski E. Horwitz P. A. Caparon M. G. (1992). Expression of protein F, the fibronectin-binding protein of *Streptococcus pyogenes* JRS4, in heterologous streptococcal and enterococcal strains promotes their adherence to respiratory epithelial cells. Infect. Immun. 60, 5119–5125. doi: 10.1128/iai.60.12.5119-5125.1992 1452345 PMC258286

[B22] Hengge R. Gründling A. Jenal U. Ryan R. Yildiz F. (2016). Bacterial signal transduction by cyclic di-GMP and other nucleotide second messengers. J. Bacteriol. 198, 15–26. doi: 10.1128/JB.00331-15 26055111 PMC4686208

[B23] Huynh T. N. Choi P. H. Sureka K. Ledvina H. E. Campillo J. Tong L.. (2016). Cyclic di-AMP targets the cystathionine beta-synthase domain of the osmolyte transporter OpuC. Mol. Microbiol. 102, 233–243. doi: 10.1111/mmi.13456 27378384 PMC5118871

[B24] Hyyryläinen H. L. Pietiäinen M. Lundén T. Ekman A. Gardemeister M. Murtomäki-Repo S.. (2007). The density of negative charge in the cell wall influences two-component signal transduction in Bacillus subtilis. Microbiol. (Reading) 153, 2126–2136. doi: 10.1099/mic.0.2007/008680-0 17600057

[B25] Kajfasz J. K. Rivera-Ramos I. Abranches J. Martinez A. R. Rosalen P. L. Derr A. M.. (2010). Two Spx proteins modulate stress tolerance, survival, and virulence in *Streptococcus mutans* . J. Bacteriol. 192, 2546–2556. doi: 10.1128/JB.00028-10 20233935 PMC2863552

[B26] Kajfasz J. K. Rivera-Ramos I. Scott-Anne K. Gregoire S. Abranches J. Lemos J. A. (2015). Transcription of Oxidative Stress Genes Is Directly Activated by SpxA1 and, to a Lesser Extent, by SpxA2 in Streptococcus mutans. J. Bacteriol. 197, 2160–2170. doi: 10.1128/JB.00118-15 25897032 PMC4455267

[B27] Kalia D. Merey G. Nakayama S. Zheng Y. Zhou J. Luo Y.. (2013). Nucleotide, c-di-GMP, c-di-AMP, cGMP, cAMP, (p)ppGpp signaling in bacteria and implications in pathogenesis. Chem. Soc. Rev. 42, 305–341. doi: 10.1039/C2CS35206K 23023210

[B28] Kamar R. Réjasse A. Jéhanno I. Attieh Z. Courtin P. Chapot-Chartier M. P.. (2017). DltX of *Bacillus thuringiensis* is essential for D-alanylation of teichoic acids and resistance to antimicrobial response in insects. Front. Microbiol. 8, 1437. doi: 10.3389/fmicb.2017.01437 28824570 PMC5541007

[B29] Kamegaya T. Kuroda K. Hayakawa Y. (2011). Identification of a *Streptococcus pyogenes* SF370 gene involved in production of c-di-AMP. Nagoya J. Med. Sci. 73, 49–57. 21614937 PMC11254362

[B30] Kang S. O. Caparon M. G. Cho K. H. (2010). Virulence gene regulation by CvfA, a putative RNase: the CvfA-enolase complex in *Streptococcus pyogenes* links nutritional stress, growth-phase control, and virulence gene expression. Infect. Immun. 78, 2754–2767. doi: 10.1128/IAI.01370-09 20385762 PMC2876558

[B31] Kang S. O. Wright J. O. Tesorero R. A. Lee H. Beall B. Cho K. H. (2012). Thermoregulation of capsule production by Streptococcus pyogenes. PloS One 7, e37367. doi: 10.1371/journal.pone.0037367 22615992 PMC3355187

[B32] Kojima N. Araki Y. Ito E. (1985). Structure of the linkage units between ribitol teichoic acids and peptidoglycan. J. Bacteriol. 161, 299–306. doi: 10.1128/jb.161.1.299-306.1985 3918002 PMC214871

[B33] Koprivnjak T. Mlakar V. Swanson L. Fournier B. Peschel A. Weiss J. P. (2006). Cation-induced transcriptional regulation of the dlt operon of *Staphylococcus aureus* . J. Bacteriol. 188, 3622–3630. doi: 10.1128/JB.188.10.3622-3630.2006 16672616 PMC1482844

[B34] Kovács M. Halfmann A. Fedtke I. Heintz M. Peschel A. Vollmer W.. (2006). A functional dlt operon, encoding proteins required for incorporation of d-alanine in teichoic acids in gram-positive bacteria, confers resistance to cationic antimicrobial peptides in Streptococcus pneumoniae. J. Bacteriol. 188, 5797–5805. doi: 10.1128/JB.00336-06 16885447 PMC1540085

[B35] Kristian S. A. Datta V. Weidenmaier C. Kansal R. Fedtke I. Peschel A.. (2005). D-alanylation of teichoic acids promotes group a streptococcus antimicrobial peptide resistance, neutrophil survival, and epithelial cell invasion. J. Bacteriol. 187, 6719–6725. doi: 10.1128/JB.187.19.6719-6725.2005 16166534 PMC1251589

[B36] Li C. Wen A. Shen B. Lu J. Huang Y. Chang Y. (2011). FastCloning: a highly simplified, purification-free, sequence- and ligation-independent PCR cloning method. BMC Biotechnol. 11, 92. doi: 10.1186/1472-6750-11-92 21992524 PMC3207894

[B37] Lin Y. Sanson M. A. Vega L. A. Shah B. Regmi S. Cubria M. B.. (2020). ExPortal and the liaFSR regulatory system coordinate the response to cell membrane stress in *Streptococcus pyogenes* . mBio 11. doi: 10.1128/mBio.01804-20 PMC7492735 32934083

[B38] Lyon W. R. Gibson C. M. Caparon M. G. (1998). A role for trigger factor and an *rgg*-like regulator in the transcription, secretion and processing of the cysteine proteinase of *Streptococcus pyogenes* . EMBO J. 17, 6263–6275. doi: 10.1093/emboj/17.21.6263 9799235 PMC1170952

[B39] Lyon W. R. Madden J. C. Levin J. C. Stein J. L. Caparon M. G. (2001). Mutation of *luxS* affects growth and virulence factor expression in *Streptococcus pyogenes* . Mol. Microbiol. 42, 145–157. doi: 10.1046/j.1365-2958.2001.02616.x 11679074

[B40] Mascher T. (2006). Intramembrane-sensing histidine kinases: a new family of cell envelope stress sensors in Firmicutes bacteria. FEMS Microbiol. Lett. 264, 133–144. doi: 10.1111/j.1574-6968.2006.00444.x 17064367

[B41] McShan W. M. Mclaughlin R. E. Nordstrand A. Ferretti J. J. (1998). Vectors containing streptococcal bacteriophage integrases for site-specific gene insertion. Meth Cell Sci. 20, 51–57. doi: 10.1023/A:1009773309163

[B42] Nakano S. Erwin K. N. Ralle M. Zuber P. (2005). Redox-sensitive transcriptional control by a thiol/disulphide switch in the global regulator, Spx. Mol. Microbiol. 55, 498–510. doi: 10.1111/j.1365-2958.2004.04395.x 15659166

[B43] Nakano S. Nakano M. M. Zhang Y. Leelakriangsak M. Zuber P. (2003). A regulatory protein that interferes with activator-stimulated transcription in bacteria. Proc. Natl. Acad. Sci. U. S. A. 100, 4233–4238. doi: 10.1073/pnas.0637648100 12642660 PMC153076

[B44] Neuhaus F. C. Baddiley J. (2003). A continuum of anionic charge: structures and functions of D-alanyl-teichoic acids in gram-positive bacteria. Microbiol. Mol. Biol. Rev. 67, 686–723. doi: 10.1128/MMBR.67.4.686-723.2003 14665680 PMC309049

[B45] Okada N. Geist R. T. Caparon M. G. (1993). Positive transcriptional control of *mry* regulates virulence in the group A streptococcus. Mol. Microbiol. 7, 893–903. doi: 10.1111/j.1365-2958.1993.tb01180.x 8483419

[B46] Pesavento C. Hengge R. (2009). Bacterial nucleotide-based second messengers. Curr. Opin. Microbiol. 12, 170–176. doi: 10.1016/j.mib.2009.01.007 19318291

[B47] Peschel A. Otto M. Jack R. W. Kalbacher H. Jung G. Götz F. (1999). Inactivation of the dlt operon in *Staphylococcus aureus* confers sensitivity to defensins, protegrins, and other antimicrobial peptides. J. Biol. Chem. 274, 8405–8410. doi: 10.1074/jbc.274.13.8405 10085071

[B48] Pham H. T. Nhiep N. T. H. Vu T. N. M. Huynh T. N. Zhu Y. Huynh A. L. D.. (2018). Enhanced uptake of potassium or glycine betaine or export of cyclic-di-AMP restores osmoresistance in a high cyclic-di-AMP *Lactococcus lactis* mutant. PloS Genet. 14, e1007574. doi: 10.1371/journal.pgen.1007574 30074984 PMC6108528

[B49] Port G. C. Cusumano Z. T. Tumminello P. R. Caparon M. G. (2017). SpxA1 and spxA2 act coordinately to fine-tune stress responses and virulence in streptococcus pyogenes. mBio 8. doi: 10.1128/mBio.00288-17 PMC5371413 28351920

[B50] Port G. C. Paluscio E. Caparon M. G. (2013). Complete Genome Sequence of emm Type 14 Streptococcus pyogenes Strain HSC5. Genome Announc. 1. doi: 10.1128/genomeA.00612-13 PMC3744678 23950122

[B51] Saar-Dover R. Bitler A. Nezer R. Shmuel-Galia L. Firon A. Shimoni E.. (2012). D-alanylation of lipoteichoic acids confers resistance to cationic peptides in group B streptococcus by increasing the cell wall density. PloS Pathog. 8, e1002891. doi: 10.1371/journal.ppat.1002891 22969424 PMC3435245

[B52] Sanson M. A. Vega L. A. Shah B. Regmi S. Cubria M. B. Horstmann N.. (2021). The liaFSR transcriptome reveals an interconnected regulatory network in group A streptococcus. Infect. Immun. 89, e0021521. doi: 10.1128/IAI.00215-21 34370508 PMC8519277

[B53] Silhavy T. J. Kahne D. Walker S. (2010). The bacterial cell envelope. Cold Spring Harb. Perspect. Biol. 2, a000414. doi: 10.1101/cshperspect.a000414 20452953 PMC2857177

[B54] Walker M. J. Barnett T. C. Mcarthur J. D. Cole J. N. Gillen C. M. Henningham A.. (2014). Disease manifestations and pathogenic mechanisms of Group A Streptococcus. Clin. Microbiol. Rev. 27, 264–301. doi: 10.1128/CMR.00101-13 24696436 PMC3993104

[B55] Watkins D. A. Johnson C. O. Colquhoun S. M. Karthikeyan G. Beaton A. Bukhman G.. (2017). Global, regional, and national burden of rheumatic heart disease 1990-2015. N Engl. J. Med. 377, 713–722. doi: 10.1056/NEJMoa1603693 28834488

[B56] Weidenmaier C. Peschel A. (2008). Teichoic acids and related cell-wall glycopolymers in Gram-positive physiology and host interactions. Nat. Rev. Microbiol. 6, 276–287. doi: 10.1038/nrmicro1861 18327271

[B57] Witte G. Hartung S. Büttner K. Hopfner K. P. (2008). Structural biochemistry of a bacterial checkpoint protein reveals diadenylate cyclase activity regulated by DNA recombination intermediates. Mol. Cell 30, 167–178. doi: 10.1016/j.molcel.2008.02.020 18439896

[B58] Woodward J. J. Iavarone A. T. Portnoy D. A. (2010). c-di-AMP secreted by intracellular *Listeria monocytogenes* activates a host type I interferon response. Science 328, 1703–1705. doi: 10.1126/science.1189801 20508090 PMC3156580

